# Good Outcome Following Attempted Resuscitation Score and Clinical Frailty Scale for Estimating Long-Term Mortality: An Ancillary Study of the CLEAR Randomized Clinical Trial

**DOI:** 10.1001/jamanetworkopen.2025.34690

**Published:** 2025-09-30

**Authors:** Samuel Kaspar Zumbrunn, Benjamin Bissmann, Sebastian Gross, Christoph Becker, Armon Arpagaus, Flavio Gössi, Philipp Schuetz, Jörg D. Leuppi, Drahomir Aujesky, Balthasar L. Hug, Thomas Peters, Arnoud J. Templeton, Stefano Bassetti, Sabina Hunziker

**Affiliations:** 1 Medical Communication and Psychosomatic Medicine, University Hospital Basel, Basel, Switzerland; 2 Medical Outpatient Department, University Hospital Basel, Basel, Switzerland; 3 Faculty of Medicine, University of Basel, Basel, Switzerland; 4 Division of Internal Medicine, University Hospital Basel, Basel, Switzerland; 5 Division of Internal Medicine, Cantonal Hospital Aarau, Aarau, Switzerland; 6 University Institute of Internal Medicine, Cantonal Hospital Baselland, Liestal, Switzerland; 7 Department of General Internal Medicine, Inselspital, Bern University Hospital, University of Bern, Bern, Switzerland; 8 Division of Internal Medicine, Cantonal Hospital Lucerne, Lucerne, Switzerland; 9 Faculty of Health Sciences and Medicine, University of Lucerne, Lucerne, Switzerland; 10 Division of Internal Medicine, St Claraspital, Basel, Switzerland; 11 Department of Medical Oncology, St Claraspital, Basel, Switzerland; 12 St Clara Research, Basel, Switzerland; 13 Post ICU Care, Department of Psychosomatic Medicine, University Hospital Basel, Basel, Switzerland

## Abstract

**Importance:**

In an aging and increasingly complex patient population, risk estimation tools are critical for optimizing clinical decision-making. The Clinical Frailty Scale (CFS) evaluates frailty and mortality risk, whereas the Good Outcome Following Attempted Resuscitation (GO-FAR) score aims to estimate survival after potential in-hospital cardiac arrest. However, their ability to estimate all-cause mortality in medical inpatients across all ages remains unclear.

**Objective:**

To assess the accuracy of the CFS and GO-FAR score in estimating long-term all-cause mortality among medical inpatients.

**Design, Setting, and Participants:**

This ancillary analysis of the CLEAR trial, a cluster randomized trial, included 2840 medical inpatients from 6 Swiss teaching hospitals.

**Intervention:**

At admission from June 1, 2019, to April 30, 2023, the CFS, GO-FAR, and Charlson Comorbidity Index (CCI) scores were recorded. Regardless of randomization group, long-term follow-up of vital status occurred between June and October 2024.

**Main Outcomes and Measures:**

The outcome was long-term all-cause mortality.

**Results:**

Among 2840 patients (mean [SD] age, 68.5 [15.8] years; 1552 [54.6%] male), 969 (34.1%) died during a mean (SD) follow-up of 3.3 (0.91) years. The GO-FAR and CFS showed good discriminatory performance for all-cause mortality with time-dependent areas under the receiver operating characteristic curve (AUROC) of 0.78 and 0.74, respectively. Combining both scores with the CCI in a combined regression model improved the overall AUROC to 0.87. Patients in the highest-risk categories had significantly increased all-cause mortality risk (GO-FAR: adjusted hazard ratio, 20.31; 95% CI, 14.71-28.06; *P* < .001; and CFS: adjusted hazard ratio, 8.69; 95% CI, 6.83-11.07; *P* < .001). Both scores demonstrated high specificity in their highest-risk categories (GO-FAR: 98.2%; CFS: 99.1%) but low sensitivity (GO-FAR: 7.8%; CFS: 9.4%). Results indicated that of 100 hospitalized patients in the highest GO-FAR category (≥14 points), approximately 69 are expected to die, whereas among those in the lower categories, approximately 67 are expected to survive (positive and negative predictive value of 69.1% and 67.3%, respectively).

**Conclusions and Relevance:**

In this ancillary study of the CLEAR trial, the GO-FAR score and CFS effectively estimated long-term all-cause mortality, particularly when combined with the CCI. These tools may help clinical decision-making, resource allocation, and advanced care planning in aging, multimorbid patient populations.

## Key Points

**Question:**

What is the performance of the Good Outcome Following Attempted Resuscitation (GO-FAR) score and Clinical Frailty Scale (CFS) to estimate long-term all-cause mortality among medical inpatients?

**Findings:**

In this ancillary analysis of the CLEAR trial among 2840 medical inpatients, the GO-FAR score and CFS showed good discriminatory performance for all-cause mortality with areas under the receiver operating characteristic curve of 0.78 and 0.74, respectively. Both scores showed high specificity but low sensitivity.

**Meaning:**

These results suggest that the GO-FAR score and CFS were effective tools for estimating long-term all-cause mortality and may thus help in clinical decision-making.

## Teaser

This ancillary analysis of the CLEAR randomized clinical trial provides an external and independent validation of the Clinical Frailty Scale and the Good Outcome Following Attempted Resuscitation score for risk estimation of all-cause mortality in medical inpatients 18 years and older.

## Introduction

In recent years, the proportion of medical inpatients with multiple chronic conditions and frailty has risen, increasing the overall complexity of care.^1,2,3,4,5^ Patients admitted to acute care facilities are known to be at a higher risk of death within the following 5 years, ranging from approximately 16.3% to 28.8% within a year of hospitalization^6,7,8,9,10^ and up to 39.7% in the long term (≥5 years),^10,11^ which is higher than in the general Swiss population.^12^ However, age alone is not a reliable predictor of mortality; instead, frailty and comorbidities are recognized as stronger, independent risk factors.^13,14,15,16,17,18^ Consequently, there is growing interest in using clinical risk scores to improve outcome prediction and guide resource allocation in an aging population.^19,20^

Among these scores, the Clinical Frailty Scale (CFS) originally developed to assess risk and estimate mortality or nursing home admission in patients 65 years and older, has since been validated for use in younger and critically ill patients.^21,22,23,24^ The CFS estimates all-cause mortality in elderly patients admitted to acute care medical and emergency departments.^14,22,25,26^ Another prognostic score for intensive care and other settings is the Good Outcome Following Attempted Resuscitation (GO-FAR) score,^27^ a prearrest score that estimates the probability of survival to hospital discharge with a good neurologic outcome after in-hospital cardiac arrest.^27,28^ Another tool that captures the comorbidity burden and the risk of a patient is the Charlson Comorbidity Index (CCI).^29^ However, the CCI serves as a general measure of comorbidity burden, often derived from coded diagnosis related group data, and is usually applied in research settings or for statistical adjustment in regression models. In contrast, the GO-FAR and CFS scores are user-friendly tools that can be quickly applied at the bedside, even in resource-limited environments, via freely accessible online calculators. Currently, it remains unclear whether combining the CCI with complementary assessments, such as the CFS and the GO-FAR score, can improve long-term mortality prognostication in hospitalized adults. As both GO-FAR and CFS are easy to apply at the bedside and highly specific for poor outcomes, integrating these tools may enhance early risk stratification, code status discussions, and resource planning beyond what comorbidity-based indexes alone offer.

The aim of this study was to provide an external and independent validation of the CFS and the GO-FAR score for estimating the risk of all-cause mortality in patients 18 years and older. This study is conducted as an ancillary analysis to the CLEAR-GUIDE project^30^ in a cohort of adult medical inpatients.

## Methods

### Study Setting

This study is an ancillary analysis of the CLEAR trial.^30^ To summarize, the CLEAR trial is a multicenter cluster randomized trial at the departments of general internal medicine of 6 Swiss teaching hospitals performed from June 1, 2019, and April 30, 2023. The CLEAR trial^30^ assessed the impact of checklist-guided communication interventions on patients’ code status preferences, satisfaction, and knowledge. The study was approved by the local ethics committee (Northwest and Central Switzerland as well as Ethics Committee Bern) and preregistered at ClinicalTrials.gov. The local ethical committee waived written consent for this follow-up study because the study involved minimal risk to participants and only collected data from routine clinical practice. This report follows the Strengthening the Reporting of Observational Studies in Epidemiology (STROBE) reporting guideline for cohort studies.^31^

### Study Design and Participants

In the main CLEAR trial, residents were randomized to receive training in either shared decision-making with a decision aid for code status discussions (intervention) or general patient-centered communication (control). To assess medical futility, we calculated the GO-FAR score and the CFS for each patient (eTables 1-3 in Supplement 1). For patients in whom cardiopulmonary resuscitation was considered futile, residents in the intervention group were trained to conduct dedicated code status discussions that included specific explanations about medical futility. As described in the original CLEAR trial publication, randomization was conducted using an interactive web-response system (SecuTrial, interActive Systems GmbH), using a computer-generated allocation scheme with block randomization and variable block sizes of 4 to 6. In the main CLEAR trial, patients were eligible regardless of their primary diagnosis. Exclusions included cognitive impairment (eg, dementia and delirium), severe mental illness, hearing impairment, language barriers, and prior trial participation. Patients deemed cardiopulmonary resuscitation futile were excluded from the main trial but included in this ancillary analysis. The study population was predominantly White, and specific data on race or ethnicity were not collected because this information is not routinely documented in medical records in Switzerland. Further details on the blinding, eligibility criteria, and communication training have previously been published.^30^

### Data Collection

Data collection occurred at baseline and during follow-up at least 1.6 years later. The GO-FAR score and CFS were assessed by a member of the study team for each patient. Additionally, the CCI was calculated by reviewing medical records at each site.^29^ Long-term follow up was conducted between June and October 2024 and consisted of screening the medical records and published obituaries for information concerning vital status.

### Outcome Measure

The main outcome was long-term, all-cause mortality. Mortality was assessed from the time of the index hospitalization to October 18, 2024, covering a follow-up period of 1.6 to 5.3 years.

### Risk Scores

Calculation of the GO-FAR score and assessment of the CFS were conducted as indicated in the original publication (eTables 1-3 in Supplement 1).^21,24,27^ The GO-FAR score was categorized according to the original publication in 4 categories (−15 to −6, above average survival; −5 to 13, average survival; 14-23, low survival; and ≥24, very low survival).^27^ We also combined the higher-risk categories (14-23 and ≥24) for further analysis. For the CFS, the results were grouped into 3 categories based on the original description and prior studies.^32,53^ CFS scores of 1 to 4 were classified as not frail, scores of 5 to 6 as moderately frail, and scores of 7 to 9 as severely frail.^21,24,32^ In addition, the CCI^29^ was calculated as a reference standard for assessing morbidity-associated mortality.^33,34,35^ We used cutoffs for severity of comorbid diseases as previously described (0, low severity; 1-2, mild severity; 3-4, moderate severity; and ≥5, high severity).^36^

### Statistical Analysis

The analyses followed a predefined analysis plan. First, we compared scores between survivors and nonsurvivors. Second, we conducted univariable and multivariable Cox proportional hazards regression analysis to study the association of the scores with all-cause mortality, calculating the hazard ratios (HRs) and 95% CIs. The multivariable models were adjusted for sex, main diagnosis at admission, study center (hospital), and randomization group. Third, we assessed the discriminatory performance of the scores using the time-dependent area under the receiver operating characteristic curve (AUROC). In addition, we evaluated the incremental prognostic value of the scores by calculation of the combined AUROCs of each score with the CCI as well as the combined AUROCs of various combinations of scores in a joint regression model. We illustrated survival after the index hospitalization and numbers at risk in each predefined category of GO-FAR score and CFS. Survival was calculated from the date of first contact to death with censoring at date of last contact during follow-up. The Kaplan-Meier method was used to estimate survival, and survival curves were compared using the log-rank test. We conducted an analysis of predefined subgroups (gender, age, and diagnosis on admission at baseline) to assess the differences between the scores’ prognostic value and all-cause mortality. We used Stata, version 15.0 (StataCorp) for all statistical analyses. A 2-sided *P* < .05 was considered statistically significant.

## Results

### Study Population

A total of 2840 patients (mean [SD] age, 68.5 [15.8] years; 1552 [54.6%] male and 1288 [45.4%] female; 2729 [96.1%] in the average or above average survival category according to the GO-FAR score) were included in this ancillary analysis and 969 patients (34.1%) died. A detailed study flowchart is shown in eFigure 1 in Supplement 1. Baseline characteristics stratified by survival status are given in Table 1. Nonsurvivors were significantly older than survivors (mean [SD] age, 74.3 [12.5] vs 65.5 [16.5] years; *P* < .001). The primary diagnoses at index hospitalization differed between the groups, with survivors having a higher percentage of ischemic heart disease (199 [10.6%] vs 45 [4.6%], *P* < .001) and nonsurvivors having a higher rate of heart failure (155 [16.0%] vs 129 [6.9%], *P* < .001) and malignant diseases (272 [28.1%] vs 149 [8.0%], *P* < .001). Clinical values also differed between groups, with nonsurvivors having higher mean (SD) CCI (6.7 [2.6] vs 3.9 [2.5], *P* < .001). Nonsurvivors were also more likely to be in the highest-risk categories of the GO-FAR scores compared with survivors.

**Table 1.** Baseline Characteristics of the Study Population

Characteristic	No. (%) of study participants^a^			*P* value
	All (N = 2840)	Survivors (n = 1871)	Nonsurvivors (n = 969)	
Sociodemographic factors at baseline				
Age, mean (SD), y	68.5 (15.8)	65.5 (16.5)	74.3 (12.5)	<.001
Sex				
Male	1552 (54.6)	994 (53.1)	558 (57.6)	.02
Female	1288 (45.4)	877 (46.9)	411 (42.4)	
Health-related factors				
Main diagnoses at baseline				
Ischemic heart disease	244 (8.6)	199 (10.6)	45 (4.6)	<.001
Heart failure	284 (10.0)	129 (6.9)	155 (16.0)	
Other cardiologic diseases	284 (10.0)	214 (11.4)	70 (7.2)	
Tumor	421 (14.8)	149 (8.0)	272 (28.1)	
Infectious diseases	340 (12.0)	235 (12.6)	105 (10.8)	
Respiratory diseases	178 (6.3)	103 (5.5)	75 (7.7)	
Gastroenterological diseases	278 (9.8)	209 (11.2)	69 (7.1)	
Metabolic diseases	130 (4.6)	96 (5.1)	34 (3.5)	
Neurological diseases	172 (6.1)	147 (7.9)	25 (2.6)	
Other	509 (17.9)	390 (20.8)	119 (12.3)	
Charlson Comorbidity Index score at baseline, mean (SD)	4.9 (2.8)	3.9 (2.5)	6.7 (2.6)	<.001
CFS score at baseline, mean (SD)	3.8 (1.5)	3.3 (1.3)	4.6 (1.5)	<.001
GO-FAR score at baseline, mean (SD)	−1.0 (7.2)	−3.1 (6.5)	2.9 (6.7)	<.001
Survival according to GO-FAR				
Very low survival category (<1%)	10 (0.4)	3 (0.2)	7 (0.7)	<.001
Low survival category (1%-3%)	101 (3.6)	32 (1.7)	69 (7.1)	
Average survival category (>3%-15%)	1813 (63.8)	1004 (53.7)	809 (83.5)	
Above average survival category (>15%)	916 (32.3)	832 (44.5)	84 (8.7)	

Abbreviations: CFS, Clinical Frailty Scale; GO-FAR, Good Outcome Following Attempted Resuscitation.

^a^ Unless otherwise indicated.

### Association Between Scores and All-Cause Mortality

Table 2 details the performance of the GO-FAR score and the CFS in estimating all-cause mortality in medical inpatients. Scores were higher in nonsurvivors compared with survivors (GO-FAR: mean [SD], 2.9 [6.7] vs −3.1 [6.5], *P* < .001; CFS: mean [SD], 4.6 [1.5] vs 3.3 [1.3], *P* < .001). For the Cox proportional hazards regression analysis, the GO-FAR score yielded a univariable HR of 1.10 per point (95% CI, 1.09-1.11; *P* < .001) and a multivariable HR of 1.10 per point (95% CI, 1.09-1.11; *P* < .001) after adjusting for sex, randomization group, principal diagnosis, and study center. The CFS yielded a univariable HR of 1.68 per point (95% CI, 1.61-1.74; *P* < .001) and a multivariable HR of 1.64 per point (95% CI, 1.57-1.71; *P* < .001). Furthermore, the risk of all-cause mortality increased for patients in the highest categories of both scores compared with the lowest categories (GO-FAR: adjusted HR, 20.31 per point in categories; 95% CI, 14.71-28.06; *P* < .001; CFS: adjusted HR, 8.69 per point in categories; 95% CI, 6.83-11.07; *P* < .001).

**Table 2.** Association of GO-FAR and CFS With All-Cause Mortality

Outcome	All (N = 2840)	Survivors (n = 1871)	Nonsurvivors (n = 969)	*P* value	Univariable analysis		Multivariable analysis	
					HR (95% CI)	*P* value	HR (95% CI)^a^	*P* value
GO-FAR score, mean (SD)	−1.0 (7.2)	−3.1 (6.5)	2.9 (6.7)	<.001	1.1 (1.09-1.11)	<.001	1.1 (1.09-1.11)	<.001
GO-FAR quartile, No. (%)								
1	728 (25.6)	673 (36.0)	55 (5.7)	<.001	1.0 [Reference]	NA	1.0 [Reference]	NA
2	791 (27.9)	591 (31.6)	200 (20.6)		3.5 (2.60-4.72)	<.001	3.24 (2.40-4.37)	<.001
3	694 (24.4)	354 (18.9)	340 (35.1)		8.27 (6.22-11.01)	<.001	6.71 (5.01-9.00)	<.001
4	627 (22.1)	253 (13.5)	374 (38.6)		12.1 (9.11-16.07)	<.001	10.31 (7.71-13.79)	<.001
GO-FAR survival category, No. (%)								
1	916 (32.3)	831 (44.4)	85 (8.8)	<.001	1.0 [Reference]	NA	1.0 [Reference]	NA
2	1814 (63.9)	1006 (53.8)	808 (83.4)		5.79 (4.63-7.25)	<.001	4.83 (3.84-6.07)	<.001
3-4	110 (3.9)	34 (1.8)	76 (7.8)		24.69 (18.06-33.75)	<.001	20.31 (14.71-28.06)	<.001
CFS score, mean (SD)	3.8 (1.5)	3.3 (1.3)	4.6 (1.5)	<.001	1.68 (1.61-1.74)	<.001	1.64 (1.57-1.71)	<.001
CFS score, No. (%)								
1-4	2132 (75.1)	1592 (85.1)	540 (55.7)	<.001	1.0 [Reference]	NA	1.0 [Reference]	NA
5-6	601 (21.2)	263 (14.1)	338 (34.9)		2.81 (2.45-3.22)	<.001	2.68 (2.33-3.08)	<.001
7-9	107 (3.8)	16 (0.9)	91 (9.4)		9.23 (7.37-11.57)	<.001	8.69 (6.83-11.07)	<.001

Abbreviations: CFS, Clinical Frailty Scale; GO-FAR, Good Outcome Following Attempted Resuscitation; HR, hazard ratio; NA, not applicable.

^a^Adjusted for cluster, principal diagnosis, sex, and study center.

Both scores showed a good discriminatory performance, with the GO-FAR score yielding an AUROC of 0.78 and the CFS yielding an AUROC of 0.74. A graphical comparison of the AUROCs is shown in eFigure 2 in Supplement 1. When combining both scores in a combined regression model, the AUROC increased to 0.85. In a further step, we additionally included the CCI in the calculation of the AUROC, which resulted in an overall AUROC of 0.87 (eFigure 2 in Supplement 1). We further calculated the discriminatory performance of different combinations of scores (eTable 4 in Supplement 1) and patient age alone because this is a factor in all aforementioned scores, which yielded an AUROC of 0.735.

The prognostic accuracy with sensitivity, specificity, positive predictive value (PPV), negative predictive value (NPV), positive log likelihood ratio [LLR], and negative LLR of the GO-FAR score and CFS at their predefined cutoffs is detailed in Table 3. The highest quartile of the GO-FAR score estimated all-cause mortality with a specificity of 86.5% and a sensitivity of 38.6%. When calculated for the predefined survival categories, the categories low survival (1%-3%) and very low survival (<1%) combined estimated all-cause mortality with a specificity of 98.2%, yet sensitivity was low at 7.8%.

**Table 3.** Performance at Different Cutoffs to Estimate All-Cause Mortality

Outcome	Mean (95% CI)					
	Sensitivity, %	Specificity, %	PPV, %	NPV, %	Positive LLR	Negative LLR
GO-FAR cutoff quartile						
>1	94.3 (92.7-95.7)	36.0 (33.8-38.2)	43.3 (41.2-45.4)	92.4 (90.3-94.3)	1.47 (1.42-1.53)	0.16 (0.12-0.21)
>2	73.7 (70.8-76.4)	67.6 (65.4-69.7)	54.0 (51.3-56.8)	83.2 (81.2-85.1)	2.27 (2.11-2.45)	0.39 (0.35-0.43)
>3	38.6 (35.5-41.7)	86.5 (84.8-88.0)	59.6 (55.7-63.5)	73.1 (71.2-75.0)	2.85 (2.48-3.28)	0.71 (0.67-0.75)
GO-FAR category cutoff						
I (≥5 points, average survival or lower)	91.2 (89.3-92.9)	44.4 (42.1-46.7)	45.9 (43.7-48.2)	90.7 (88.7-92.5)	1.64 (1.57-1.72)	0.20 (0.16-0.24)
II (≥14 points, low or very low survival)	7.8 (6.2-9.7)	98.2 (97.5-98.7)	69.1 (59.6-77.6)	67.3 (65.5-69.0)	4.32 (2.90-6.42)	0.94 (0.92-0.96)
CFS category cutoff						
I (≥5)	44.3 (41.1-47.5)	85.1 (83.4-86.7)	60.6 (56.9-64.2)	74.7 (72.8-76.5)	2.97 (2.61-3.38)	0.65 (0.62-0.69)
II (≥7)	9.4 (7.6-11.4)	99.1 (98.6-99.5)	85.0 (76.9-91.2)	67.9 (66.1-69.6)	10.98 (6.49-18.58)	0.91 (0.90-0.93)

Abbreviations: CFS, Clinical Frailty Scale; GO-FAR, Good Outcome Following Attempted Resuscitation; LLR, likelihood ratio; NPV, negative predictive value; PPV, positive predictive value.

We also calculated PPV and NPV. The data indicated that of 100 hospitalized patients in the highest GO-FAR category (≥14 points), approximately 69 were estimated to die within the following 3 to 4 years. In contrast, among those in the lower categories (<14 points), approximately 67 were expected to survive during the same period (PPV and NPV of 69.1% and 67.3%, respectively). When categorized as high risk (CFS score, 7-9) vs lower risk (CFS score, 1-6), the sensitivity and specificity were 99.1% and 9.4%, respectively. For these highest-risk categories, the positive LLR and negative LLR were 4.32 and 0.94 for the GO-FAR score and 10.98 and 0.91 for the CFS, respectively. Kaplan-Meier survival curves with the corresponding numbers at risk, stratified by quartiles for the GO-FAR score and the predefined categories for the CFS, are presented in the Figure.

**Figure.**
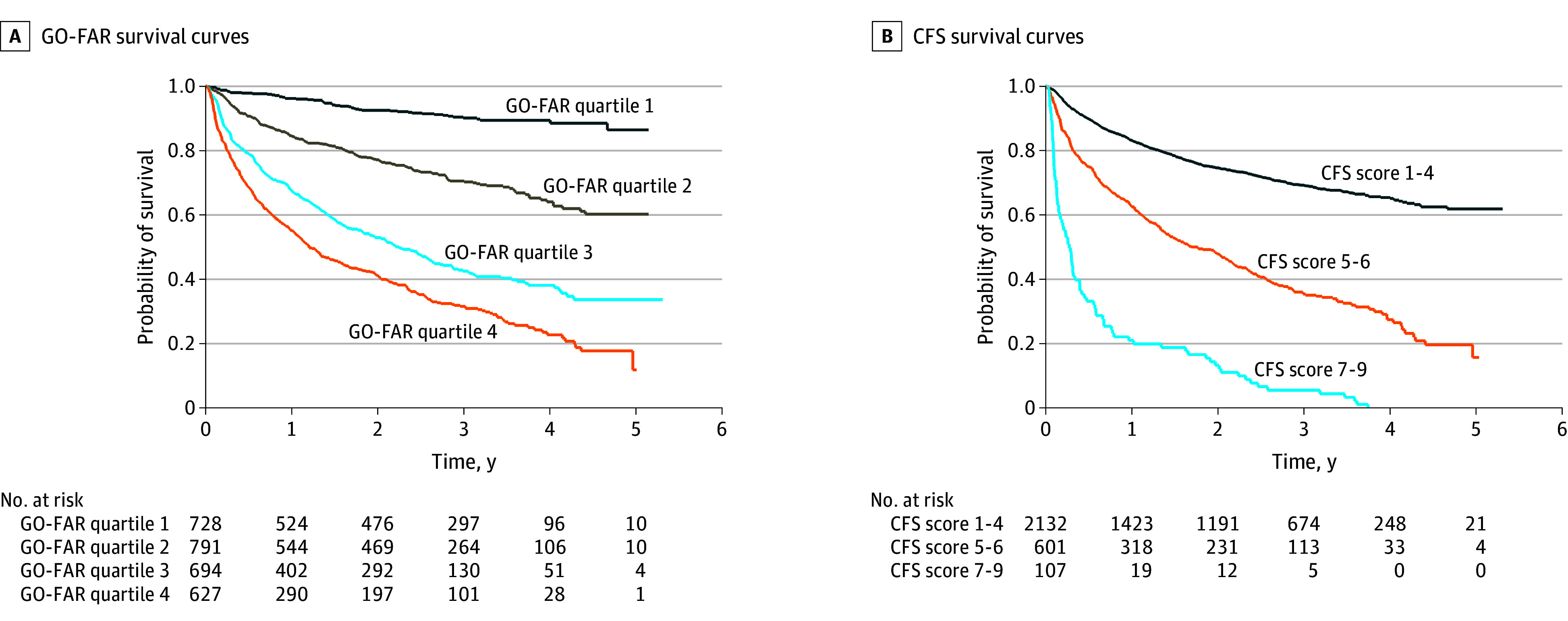
Kaplan-Meier Survival Estimates With Number at Risk for Good Outcome Following Attempted Resuscitation (GO-FAR) Score Quartiles and Clinical Frailty Scale (CFS) Categories

In addition, we conducted subgroup analyses to assess the differences between the scores’ prognostic value and all-cause mortality in specific patient groups (eFigure 3 in Supplement 1). For the GO-FAR score, cardiologic principal diagnosis and absence of metabolic or infectious principal diagnosis were significantly associated with better score performance. Associations were similar for the CFS, with absence of oncologic principal diagnosis being associated with better performance. In addition, we calculated the logistical regression models for all scores (eTables 5 and 6 in Supplement 1) and the performance of the CCI (eTables 7 and 8 in Supplement 1).

## Discussion

This study aimed to validate 1 prearrest score (GO-FAR score) and 1 frailty scale (CFS) for estimating risk of all-cause mortality in a prospective cohort of medical inpatients aged 18 to 99 years and followed up for up to 5.3 years. Both scores were independent factors associated with all-cause mortality and stayed robust after adjusting for sex, cluster, principal diagnosis, and study center. The GO-FAR score and CFS showed acceptable prognostic performance in our cohort, notably outperforming patient age alone, which is a component of all the mentioned scores. Sensitivity and NPV were particularly high in the highest-risk categories for both scores, albeit at the cost of low specificity. Adding the CCI further enhanced prognostic performance of both scores.

Overall, all-cause mortality in our cohort was 34.1%. These data are in line with previous, international research that reported similar long-term survival rates in medical inpatients, ranging from 28.8% within a year of hospital admission up to 39.7% during 5 years.^6,7,8,9,10,11^ To our knowledge, this is the first validation of the GO-FAR score for the outcome of all-cause mortality in medical inpatients. The GO-FAR score was developed in 2013 using data from the US Get With the Guidelines–Resuscitation Registry and aims to estimate survival to hospital discharge with a favorable neurologic outcome in the event of an in-hospital cardiac arrest. The discriminatory performance in the internal validation cohort was good, with an AUROC of 0.78.^27^ External validation studies^37,38,39,40,41,42,43^ of the GO-FAR score have demonstrated its strong prognostic performance, achieving an AUROC range of 0.68 to 0.85 in estimating its validated outcomes. In 2 recent meta-analyses,^28,44^ the pooled AUROC was 0.78. Although validated for a different outcome, these results align with the score’s good performance in estimating long-term all-cause mortality in our cohort in which the GO-FAR score achieved an AUROC of 0.78 for estimating long-term all-cause mortality. The similarity of these findings may not be coincidental. The GO-FAR score incorporates 13 factors, many of which reflect underlying conditions or chronic illnesses, such as the neurologic condition at admission, metastatic or hematologic cancer, residence in a skilled nursing facility, and renal insufficiency or dialysis.^27^ Previous research^45,46,47,48^ has shown these factors to be independent risk factors for all-cause mortality. Hence, this may explain the similar performance of the GO-FAR score in our cohort. However, further research is needed to confirm this hypothesis.

Similarly, the CFS demonstrated performance consistent with previous validation studies^14,26,49^ evaluating all-cause mortality. Theou et al^26^ reported an AUROC of 0.73 for 2-year and 0.70 for 5-year all-cause mortality in noninstitutionalized patients aged 50 to 104 years. Rueegg et al^14^ observed an AUROC of 0.77 for the CFS’ performance in predicting 1-year all-cause mortality in a cohort of emergency department patients 65 years and older. Chong et al^49^ found an AUROC of 0.80 in geriatric patients 65 years and older. In our cohort, which included patients aged 18 to 99 years and followed for up to 5 years, the CFS demonstrated an AUROC of 0.74. Furthermore, the CFS was significantly associated with all-cause mortality. This association was even more pronounced in patients classified in the severe frailty category (CFS score, 7-9), where the adjusted HR increased significantly. Consistent with our findings, previous work has reported similar associations, with higher CFS categories (CFS score, ≥5) demonstrating increased all-cause mortality risk.^49^ However, the lack of standardized cutoffs across studies for defining frailty vs nonfrailty complicates direct comparisons among categories.

Despite being developed for a different outcome, the GO-FAR score demonstrates potential for estimating all-cause mortality and may help for clinical decision-making in medical inpatients. Similarly, the CFS is a practical tool for assessing frailty and identifying high-risk patients. Both scores are simple to compute using free online calculators.^50,51^ Both scores are highly specific in identifying individuals at elevated risk of all-cause mortality, and their strong NPVs further support their usefulness in identifying patients likely to survive. Notably, the highest-risk categories were associated with significantly elevated adjusted HRs, underscoring their capacity to delineate a particularly at-risk subgroup. Therefore, the principal utility of these tools lies in identifying patients at high risk of death.

However, clinicians should be aware of potential biases, including the risk of a self-fulfilling prophecy in prognostication. This describes how the reporting of a poor prognosis, among other factors by risk scores, at an early stage of the treatment process can lead to a treatment alteration or discontinuation, which results in a higher incidence of poor outcomes.^52^ Importantly, in our cohort, physicians did not have information on either calculated score and an influence seems thus unlikely.

### Strengths and Limitations

Strengths of our study include the large patient population of all ages from 6 Swiss teaching hospitals. Furthermore, the medical team involved in the treatment decisions was not aware of the calculated scores, therefore limiting their impact on clinical decision-making. Still, this study has limitations. First, the study only included medical inpatients from 6 Swiss teaching hospitals and excluded patients with dementia or delirium—both significant factors associated with mortality and integral parts of the GO-FAR and CFS. This limits the applicability of the findings to other settings. Second, changes in the medical condition of the patients were not subsequently assessed and included in the final analysis. Third, vital status was assessed by screening the medical records and published obituaries. It is thus possible that some deaths went undetected.

## Conclusions

In this ancillary analysis of a multicenter cluster randomized trial, the GO-FAR score and CFS showed a good performance in risk estimation for long-term all-cause mortality. The GO-FAR score showed the best discriminatory performance, and this performance was further enhanced by additionally adding the CCI. Given the growing importance of initiating code status discussions early during hospitalization, both scores are increasingly being applied in this context. Therefore, the ability to use already calculated scores for the additional estimation of all-cause mortality may prove highly valuable in supporting timely and informed decision-making. Because both the GO-FAR score and the CFS scores are simple to use, easily available, and rely on readily available clinical information, they have the potential to be applied across a broad range of hospital settings. They might thus help to better stratify patients at risk and could improve optimal allocation of resources. Furthermore, because these tools are based on clinical variables rather than health system–specific data, the findings may be applicable to other settings. However, validation in other populations and health care settings is needed to confirm generalizability.

## Notes

Supplement 1. **eFigure 1.** Flowchart **eFigure 2.** Comparison of ROC curves of the GO-FAR (A), CFS (B), and combined time dependent AUROC of all scores combined with the CCI (C) **eFigure 3.** Subgroup analysis of the GO-FAR score (A) and the CFS (B) for primary endpoint mortality **eTable 1.** Description of the GO-FAR Score **eTable 2.** Description of the Clinical Frailty Scale **eTable 3.** Description of the CCI **eTable 4.** Comparison of AUROC Values for Individual and Combined Risk Scores **eTable 5.** Association and Prognostic Performance of the GO-FAR with All-Cause Mortality (logistic regression model) **eTable 6.** Association and Prognostic Performance of the CCI with All-Cause Mortality (logistic regression model) **eTable 7**. Association and Prognostic Performance of the CCI with All-Cause Mortality **eTable 8.** Performance of CCI at different Cut-Offs to predict All-Cause-Mortality
eReferences

Supplement 2.
Data Sharing Statement
