# Supplementary material for: Good Outcome Following Attempted Resuscitation Score and Clinical Frailty Scale for Estimating Long-Term Mortality: An Ancillary Study of the CLEAR Randomized Clinical Trial
Source: JAMA Netw Open. 2025 Sep 30;8(9):e2534690. doi: 10.1001/jamanetworkopen.2025.34690 (PMC12485637; doi:10.1001/jamanetworkopen.2025.34690)
Supplement: Supplement 1. — eFigure 1. Flowchart eFigure 2. Comparison of ROC curves of the GO-FAR (A), CFS (B), and combined time dependent AUROC of all scores combined with the CCI (C) eFigure 3. Subgroup analysis of the GO-FAR score (A) and the CFS (B) for primary endpoint mortality eTable 1. Description of the GO-FAR Score eTable 2. Description of the Clinical Frailty Scale eTable 3. Description of the CCI eTable 4. Comparison of AUROC Values for Individual and Combined Risk Scores eTable 5. Association and Prognostic Performance of the GO-FAR with All-Cause Mortality (logistic regression model) eTable 6. Association and Prognostic Performance of the CCI with All-Cause Mortality (logistic regression model) eTable 7. Association and Prognostic Performance of the CCI with All-Cause Mortality eTable 8. Performance of CCI at different Cut-Offs to predict All-Cause-Mortality eReferences [file jamanetwopen-e2534690-s001.pdf]

Supplemental Online Content

Zumbrunn SK, Bissmann B, Gross S, et al. Good Outcome Following Attempted Resuscitation score and Clinical Frailty Scale for long-term mortality prognostication: an ancillary study of the CLEAR randomized clinical trial. *JAMA Netw Open*. 2025;8(9):e2534690. doi:10.1001/jamanetworkopen.2025.34690

eFigure 1. Flowchart

eFigure 2. Comparison of ROC curves of the GO-FAR (A), CFS (B), and combined time dependent AUROC of all scores combined with the CCI (C)

eFigure 3. Subgroup analysis of the GO-FAR score (A) and the CFS (B) for primary endpoint mortality

eTable 1. Description of the GO-FAR Score[1]

eTable 2. Description of the Clinical Frailty Scale[2,3]

eTable 3. Description of the CCI[4]

eTable 4. Comparison of AUROC Values for Individual and Combined Risk Scores

eTable 5. Association and Prognostic Performance of the GO-FAR with All-Cause Mortality (logistic regression model**)**

eTable 6. Association and Prognostic Performance of the CCI with All-Cause Mortality (logistic regression model)

eTable 7. Association and Prognostic Performance of the CCI with All-Cause Mortality

eTable 8. Performance of CCI at different Cut-Offs to predict All-Cause-Mortality

eReferences

This supplemental material has been provided by the authors to give readers additional information about their work.

© 2025 Zumbrunn SK et al. *JAMA Network Open*.

eFigure 1. Flowchart

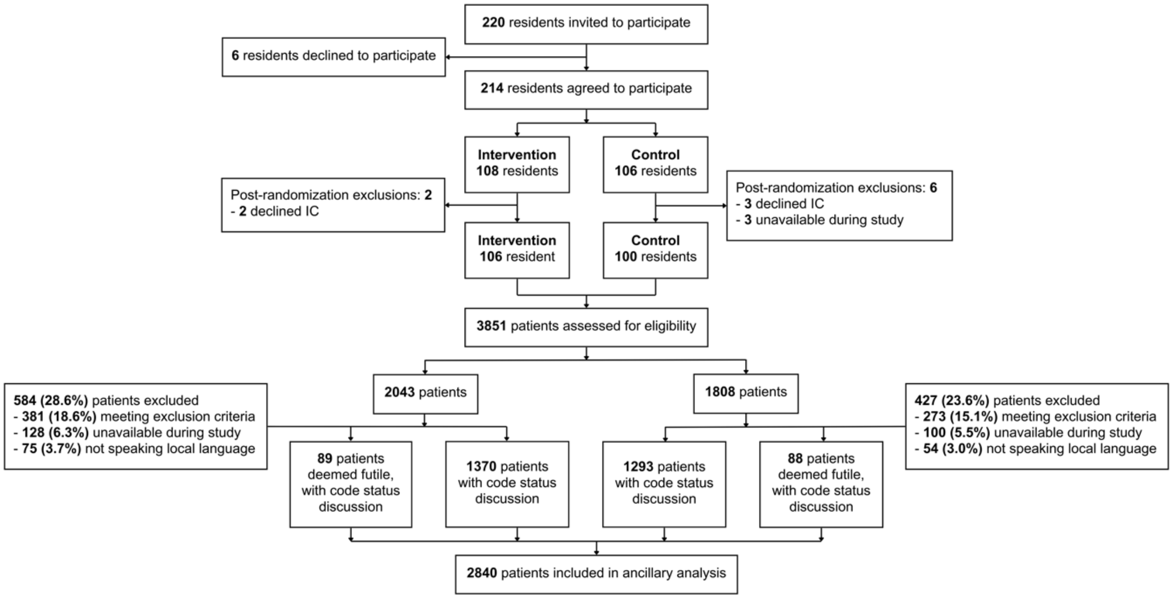

© 2025 Zumbrunn SK et al. *JAMA Network Open*.

eFigure 2. Comparison of ROC curves of the GO-FAR (A), CFS (B), and combined time dependent AUROC of all scores combined with the CCI (C)

eFigure 3a

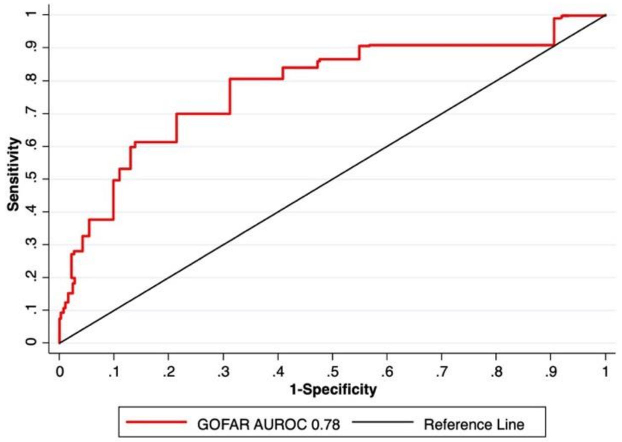

eFigure 3b

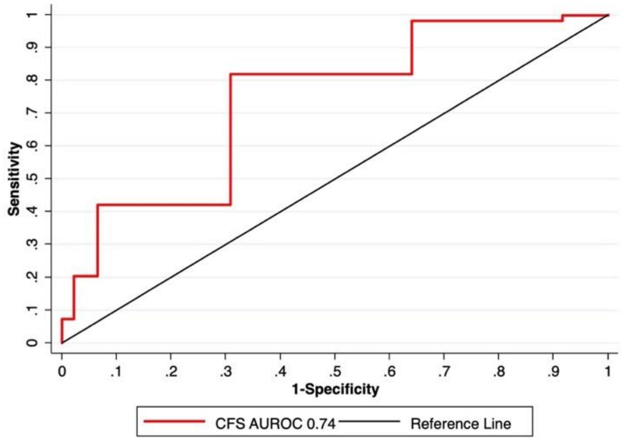

eFigure 3c

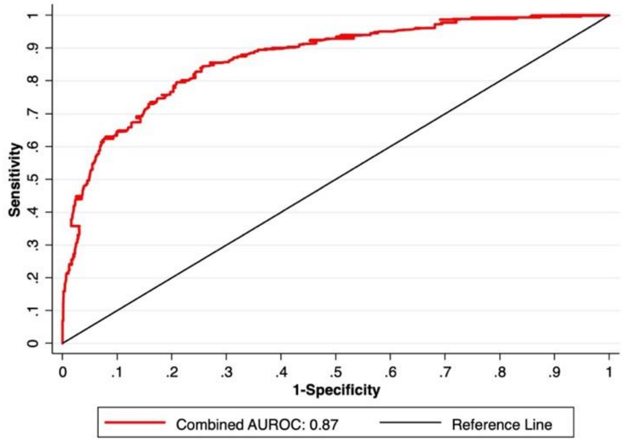

Abbreviations: AUROC Area under the receiver operating characteristic curve; **CFS** Clinical Frailty Scale; **CCI** Charlson Comorbidity Index; **GO-FAR** GO-FAR (Good Outcome Following Attempted Resuscitation) Score.

© 2025 Zumbrunn SK et al. *JAMA Network Open*.

eFigure 3. Subgroup analysis of the GO-FAR score (A) and the CFS (B) for primary endpoint mortality

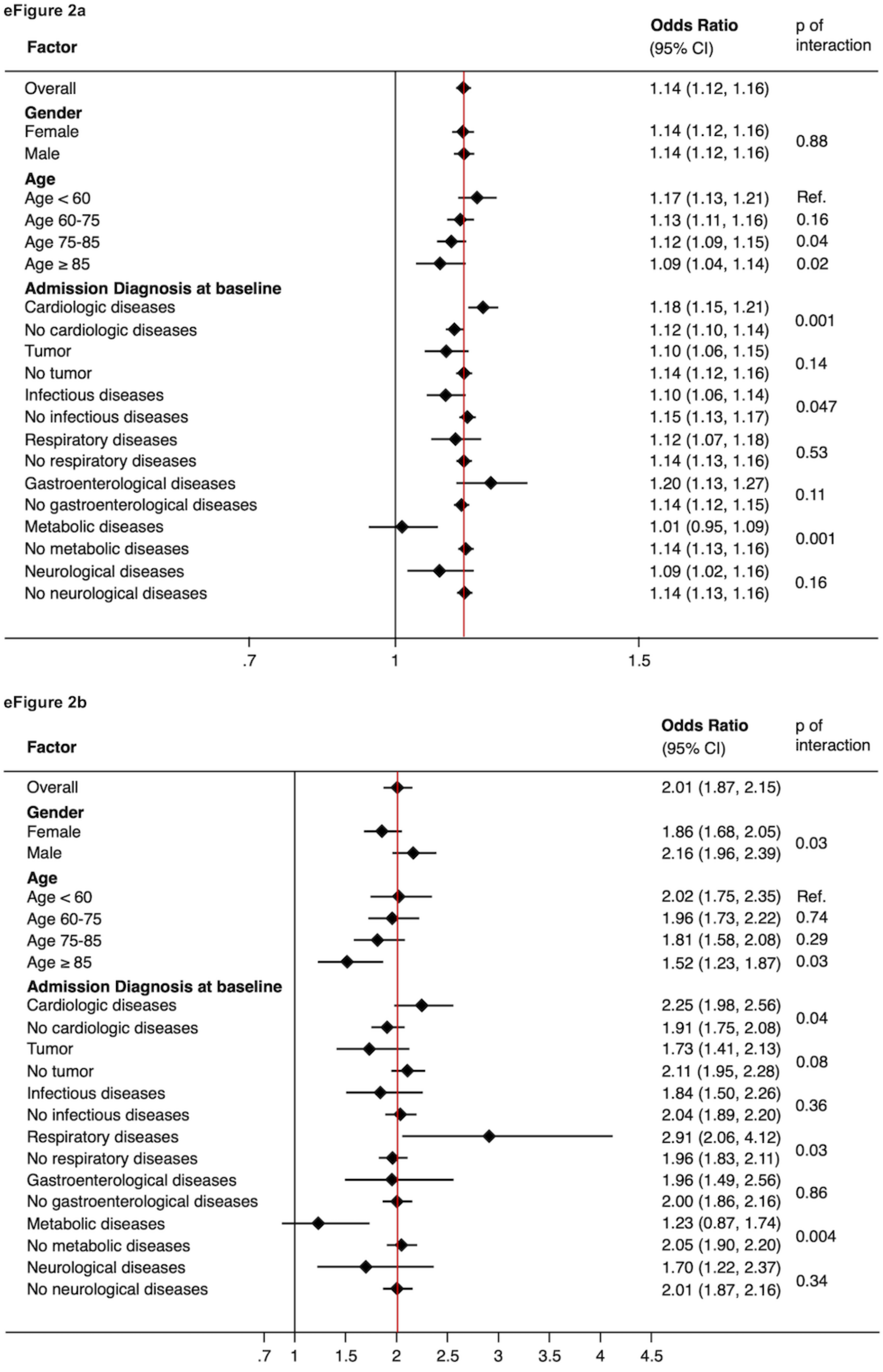

© 2025 Zumbrunn SK et al. *JAMA Network Open*.

Data is presented as univariable odds ratio (OR) and 95% confidence interval (95% CI). Abbreviations: **GO-FAR** GO-FAR (Good Outcome Following Attempted Resuscitation) Score; **CFS** Clinical Frailty Scale.

© 2025 Zumbrunn SK et al. *JAMA Network Open*.

eTable 1. Description of the GO-FAR Score[1]

Outcome predicted	Variable	Score calculation	Probability categories for primary outcome in original publication
Good neurological outcome (CPC 1)	CPC 1 at admission	-15	≥24: very low
	Major trauma	10	14 to 23: low
	Acute stroke	8	-5 to 13: average
	Metastatic or hematologic cancer	7	-15 to -6: above average
	Septicaemia	7	
	Medical noncardiac diagnosis	7	
	Hepatic insufficiency	6	
	Admitted from skilled nursing facility	6	
	Hypotension or hypoperfusion	5	
	Renal insufficiency or dialysis	4	
	Respiratory insufficiency	4	
	Pneumonia	1	
	Age 70-74 years	2	
	Age 75-79 years	5	
	Age 80-84 years	6	
	Age ≥85 years	11	

Abbreviations: **GO-FAR** GO-FAR (Good Outcome Following Attempted Resuscitation) Score; **CPC** Cerebral Performance Category.

© 2025 Zumbrunn SK et al. *JAMA Network Open*.

eTable 2. Description of the Clinical Frailty Scale[2,3]

Scale	Description:
**1**	Patients that are robust, active, energetic, motivated and fit. This group of patients commonly exercises regularly and is in the most fit group for their age.
**2**	People without active disease symptoms, but they are less fit than category 1. Occasional exercise or activity.
**3**	People with well controlled medical issues, without regular activity beyond routine walking
**4**	Symptoms often limit activities, yet this group is not dependent on others for daily help. Being slowed up +/- being tired during the day is a common issue.
**5**	Slowing is more evident and limits higher order IADLs (instrumental activities of daily living), such as shopping.
**6**	This group needs help with all outside activities and with housekeeping. Help is needed for bathing or dressing and this group often has problems with stairs.
**7**	Complete dependence on others for daily care, yet stable and not at high risk of dying within 6 months.
**8**	Complete dependence on others for daily care and unable to recover from a mild illness. Approaching the end of life.
**9**	Approaching the end of life, life expectancy below 6 months, not otherwise severely frailty

Abbreviations: **CFS** Clinical Frailty Scale.

© 2025 Zumbrunn SK et al. *JAMA Network Open*.

eTable 3. Description of the CCI[4]

**Factor**	**Weight**
Myocardial Infarct	1
Congestive heart failure	1
Peripheral vascular disease	1
Cerebrovascular disease	1
Dementia	1
Chronic pulmonary disease	1
Connective tissue disease	1
Ulcer disease	1
Mild liver disease	1
Diabetes	1
Hemiplegia	2
Moderate or severe renal disease	2
Diabetes with end organ damage	2
Any tumour	2
Leukemia	2
Lymphoma	2
Moderate or severe liver disease	3
Metastatic solid tumor	6
AIDS	6
Age: 1 point for each decade > 40 years, 0 point If below 50 years, 1 point 50-59 years, etc.	

**Severity:**<br>1 = not ill,<br>2 = mildly ill<br>3 = moderately ill<br>4 = severely ill<br>5 = moribund.	**Calculation:**<br>Sum all scored points<br><br>10-year survival rate = $0.983^{e(CCI \times 0.9)}$

Abbreviations: **CCI** Charlson Comorbidity Index..

© 2025 Zumbrunn SK et al. *JAMA Network Open*.

eTable 4. Comparison of AUROC Values for Individual and Combined Risk Scores

Factors	AUC
**GO-FAR**	0.78
**CFS**	0.74
**CCI**	0.78
**Combined GO-FAR, CFS, CCI**	0.87
**Combined GO-FAR, CFS**	0.85
**Combined GO-FAR, CCI**	0.85
**Combined CFS, CCI**	0.86

Abbreviations: **AUROC** Area under the receiver operating characteristic curve; **CFS** Clinical Frailty Scale; **CCI** Charlson Comorbidity Index; **GO-FAR** GO-FAR (Good Outcome Following Attempted Resuscitation) Score.

© 2025 Zumbrunn SK et al. *JAMA Network Open*.

eTable 5. Association and Prognostic Performance of the GO-FAR with All-Cause Mortality (logistic regression model)

Factor	All	Survivors	Non-survivors	p-value	Univariable OR (95% CI)	p-value	Multivariable OR* (95% CI)	p-value
**N, n (%)**	2840	1871	969		2840		2840	
**GO-FAR, mean (SD)**	-1.0 (7.2)	-3.1 (6.5)	2.9 (6.7)	<0.001	1.14 (1.12, 1.16)	<0.001	1.14 (1.12, 1.15)	<0.001
**GO-FAR quartile 1, n (%)**	728 (25.6)	673 (36.0)	55 (5.7)	<0.001	(Ref.)		(Ref.)	
**GO-FAR quartile 2, n (%)**	791 (27.9)	591 (31.6)	200 (20.6)		4.14 (3.01, 5.69)	<0.001	3.98 (2.87, 5.53)	<0.001
**GO-FAR quartile 3, n (%)**	694 (24.4)	354 (18.9)	340 (35.1)		11.75 (8.6, 16.06)	<0.001	9.12 (6.58, 12.63)	<0.001
**GO-FAR quartile 4, n (%)**	627 (22.1)	253 (13.5)	374 (38.6)		18.09 (13.16, 24.86)	<0.001	15.71 (11.28, 21.88)	<0.001
**GO-FAR Survival Category 1 (Above average), n (%)**	916 (32.3)	831 (44.4)	85 (8.8)	<0.001	(Ref.)		(Ref.)	
**GO-FAR Survival Category 2 (Average), n (%)**	1814 (63.9)	1006 (53.8)	808 (83.4)		7.85 (6.17, 10)	<0.001	6.67 (5.18, 8.58)	<0.001
**GO-FAR Survival Categories 3-4 (Low And Very Low), n (%)**	110 (3.9)	34 (1.8)	76 (7.8)		21.85 (13.77, 34.68)	<0.001	19.73 (12.03, 32.35)	<0.001
**CFS, mean (SD)**	3.8 (1.5)	3.3 (1.3)	4.6 (1.5)	<0.001	2.01 (1.87, 2.15)	<0.001	2.03 (1.88, 2.19)	<0.001
**CFS 1-4, n (%)**	2132 (75.1)	1592 (85.1)	540 (55.7)	<0.001	(Ref.)		(Ref.)	
**CFS 5-6, n (%)**	601 (21.2)	263 (14.1)	338 (34.9)		3.79 (3.14, 4.57)	<0.001	3.99 (3.26, 4.9)	<0.001
**CFS 7-9, n (%)**	107 (3.8)	16 (0.9)	91 (9.4)		16.77 (9.77, 28.78)	<0.001	17.5 (9.86, 31.08)	<0.001

eTable 2a. Association and Prognostic Performance of GO-FAR and CFS with All-Cause Mortality
Data presented as n (%) or mean (95% CI) unless otherwise specified. *adjusted for cluster, principal diagnosis, sex, study center. Abbreviations: **OR,** Odds Ratio; **SD**, standard deviation; **CI**, confidence interval; **SD**, standard deviation; **n**, number. **GO-FAR** GO-FAR (Good Outcome Following Attempted Resuscitation) Score; **CFS** Clinical Frailty Scale; **AUC** Area Under The Curve.

© 2025 Zumbrunn SK et al. *JAMA Network Open*.

eTable 6. Association and Prognostic Performance of the CCI with All-Cause Mortality (logistic regression model)

Factor	All	Survivors	Non-survivors	p-value	Univariable OR (95% CI)	p-value	Multivariable OR* (95% CI)	p-value
**N**	2840	1871	969		2840		2840	
**CCI, mean (SD)**	4.9 (2.84)	3.9 (2.5)	6.7 (2.6)	<0.001	1.54 (1.48, 1.6)	<0.001	1.49 (1.43, 1.55)	<0.001
**CCI Class I (0 = low), n (%)**	198 (7.0)	193 (10.3)	5 (0.5)	<0.001	(Ref.)		(Ref.)	
**CCI Class II (1-2 = mild), n (%)**	391 (13.8)	358 (19.1)	33 (3.4)		3.56 (1.37, 9.26)	0.009	3.07 (1.17, 8.04)	0.023
**CCI Class III (3-4 = moderate), n (%)**	721 (25.4)	589 (31.5)	132 (13.6)		8.65 (3.49, 21.44)	<0.001	7.11 (2.85, 17.72)	<0.001
**CCI Class IV (≥ 5 = severe), n (%)**	1530 (53.9)	731 (39.1)	799 (82.5)		42.19 (17.27, 103.1)	<0.001	31.38 (12.76, 77.2)	<0.001
**CCI Quartile 1, n (%)**	915 (32.2)	827 (44.2)	88 (9.1)	<0.001	(Ref.)		(Ref.)	
**CCI Quartile 2, n (%)**	814 (28.7)	583 (31.2)	231 (23.8)		3.72 (2.85, 4.87)	<0.001	3.48 (2.64, 4.58)	<0.001
**CCI Quartile 3, n (%)**	634 (22.3)	325 (17.4)	309 (31.9)		8.94 (6.83, 11.7)	<0.001	7.75 (5.86, 10.25)	<0.001
**CCI Quartile 4, n (%)**	477 (16.8)	136 (7.3)	341 (35.2)		23.56 (17.52, 31.69)	<0.001	18.5 (13.56, 25.23)	<0.001

eTable 2a. Association and Prognostic Performance of CCI with All-Cause Mortality
Data presented as n (%) or mean (95% CI) unless otherwise specified. *adjusted for cluster, principal diagnosis, sex, study center. Abbreviations: **OR,** Odds Ratio; **SD**, standard deviation; **CI**, confidence interval; **SD**, standard deviation; **n**, number. **CCI** Charlson Comorbidity Index; **AUC** Area Under The Curve.

© 2025 Zumbrunn SK et al. *JAMA Network Open*.

eTable 7. Association and Prognostic Performance of the CCI with All-Cause Mortality

Factor	All	Survivors	Non-survivors	p-value	Univariable HR (95% CI)	p-value	Multivariable HR* (95% CI)	p-value	Time dependent AUC
**N**	2840	1871	969		2840		2840		
**CCI, mean (SD)**	4.9 (2.84)	3.9 (2.5)	6.7 (2.6)	<0.001	1.34 (1.31, 1.37)	<0.001	1.29 (1.26, 1.32)	<0.001	0.78
**CCI Class I (0 = low), n (%)**	198 (7.0)	193 (10.3)	5 (0.5)	<0.001	(Ref.)		(Ref.)		
**CCI Class II (1-2 = mild), n (%)**	391 (13.8)	358 (19.1)	33 (3.4)		3.37 (1.32, 8.64)	0.011	3.08 (1.2, 7.9)	0.019	
**CCI Class III (3-4 = moderate), n (%)**	721 (25.4)	589 (31.5)	132 (13.6)		7.91 (3.24, 19.33)	<0.001	6.76 (2.76, 16.53)	<0.001	
**CCI Class IV (≥ 5 = severe), n (%)**	1530 (53.9)	731 (39.1)	799 (82.5)		28.31 (11.75, 68.22)	<0.001	21.37 (8.85, 51.64)	<0.001	
**CCI Quartile 1, n (%)**	915 (32.2)	827 (44.2)	88 (9.1)	<0.001	(Ref.)		(Ref.)		
**CCI Quartile 2, n (%)**	814 (28.7)	583 (31.2)	231 (23.8)		3.29 (2.58, 4.21)	<0.001	3.00 (2.35, 3.85)	<0.001	
**CCI Quartile 3, n (%)**	634 (22.3)	325 (17.4)	309 (31.9)		6.68 (5.27, 8.47)	<0.001	5.63 (4.43, 7.17)	<0.001	
**CCI Quartile 4, n (%)**	477 (16.8)	136 (7.3)	341 (35.2)		11.93 (9.42, 15.1)	<0.001	9.15 (7.18, 11.67)	<0.001	

Data presented as n (%) or mean (95% CI) unless otherwise specified. *adjusted for cluster, principal diagnosis, sex, study center. Abbreviations: **HR,** Hazard Ratio; **SD**, standard deviation; **CI**, confidence interval; **SD**, standard deviation; **n**, number. **CCI** Charlson Comorbidity Index; **AUC** Area Under The Curve.

© 2025 Zumbrunn SK et al. *JAMA Network Open*.

eTable 8. Performance of CCI at different Cut-Offs to predict All-Cause-Mortality

Factor	Sensitivity (%)	Specificity (%)	PPV (%)	NPV (%)	LLR+	LLR-
**CCI Class Cutoff I (≥ 1 point)**	99.5 (98.8, 99.8)	10.3 (9.0, 11.8)	36.5 (34.6, 38.4)	97.5 (94.2, 99.2)	1.11 (1.09, 1.13)	0.05 (0.02, 0.12)
**CCI Class Cutoff II (≥ 3 points)**	96.1 (94.7, 97.2)	29.4 (27.4, 31.6)	41.4 (39.3, 43.4)	93.5 (91.3, 95.4)	1.36 (1.32, 1.41)	0.13 (0.10, 0.18)
**CCI Class Cutoff III (≥ 5 points)**	82.5 (79.9, 84.8)	60.9 (58.7, 63.1)	52.2 (49.7, 54.8)	87.0 (85.1, 88.8)	2.11 (1.98, 2.25)	0.29 (0.25, 0.33)
**CCI Cutoff Quartile > 1**	90.9 (88.9, 92.7)	44.2 (41.9, 46.5)	45.8 (43.5, 48.0)	90.4 (88.3, 92.2)	1.63 (1.56, 1.70)	0.21 (0.17, 0.25)
**CCI Cutoff Quartile > 2**	67.1 (64.0, 70.0)	75.4 (73.3, 77.3)	58.5 (55.5, 61.4)	81.6 (79.6, 83.4)	2.72 (2.49, 2.98)	0.44 (0.40, 0.48)
**CCI Cutoff Quartile > 3**	35.2 (32.2, 38.3)	92.7 (91.5, 93.9)	71.5 (67.2, 75.5)	73.4 (71.6, 75.2)	4.84 (4.03, 5.81)	0.70 (0.67, 0.73)

Data presented as mean (95% CI) unless otherwise specified. Abbreviations: **CCI** Charlson Comorbidity Index; **LLR+** Positive likelihood ratio; **LLR-** Negative likelihood ratio; **NPV** Negative predictive value; **PPV** Positive predictive value.

© 2025 Zumbrunn SK et al. *JAMA Network Open*.

eReferences

1. Ebell MH, Jang W, Shen Y, Geocadin RG. Development and validation of the Good Outcome Following Attempted Resuscitation (GO-FAR) score to predict neurologically intact survival after in-hospital cardiopulmonary resuscitation. JAMA Intern Med 2013;173(20):1872-8. (In eng). DOI: 10.1001/jamainternmed.2013.10037.
2. Rockwood K, Song X, MacKnight C, et al. A global clinical measure of fitness and frailty in elderly people. Cmaj 2005;173(5):489-95. (In eng). DOI: 10.1503/cmaj.050051.
3. Rockwood K, Theou O. Using the Clinical Frailty Scale in Allocating Scarce Health Care Resources. Can Geriatr J 2020;23(3):210-215. (In eng). DOI: 10.5770/cgj.23.463.
4. Charlson ME, Pompei P, Ales KL, MacKenzie CR. A new method of classifying prognostic comorbidity in longitudinal studies: development and validation. J Chronic Dis 1987;40(5):373-83. (In eng). DOI: 10.1016/0021-9681(87)90171-8.

© 2025 Zumbrunn SK et al. *JAMA Network Open*.
