# Supplementary material for: Good Outcome Following Attempted Resuscitation Score and Clinical Frailty Scale for Estimating Long-Term Mortality: An Ancillary Study of the CLEAR Randomized Clinical Trial
Source: JAMA Netw Open. 2025 Sep 30;8(9):e2534690. doi: 10.1001/jamanetworkopen.2025.34690 (PMC12485637; doi:10.1001/jamanetworkopen.2025.34690)
Supplement: Supplement 2. — Data Sharing Statement [file jamanetwopen-e2534690-s002.pdf]

Data Sharing Statement

Zumbrunn. Good Outcome Following Attempted Resuscitation Score and Clinical Frailty Scale for Estimating Long-Term Mortality. *JAMA Netw Open*. Published September 30, 2025. doi:10.1001/jamanetworkopen.2025.34690

Data
Data available: No

Additional Information
Explanation for why data not available: Data available upon request
